# Perforated pre-pyloric ulcer in the gastric remnant over a decade after Roux-en-Y gastric bypass: A case report

**DOI:** 10.1016/j.ijscr.2023.108888

**Published:** 2023-10-01

**Authors:** Bianca Marquez, Emmanuel Luciano, Ryan Cohen, Christina Maser, Matthew Hubbard

**Affiliations:** a Department of Surgery, Central Michigan University College of Medicine, United States of America; b Department of Surgery, UC Davis Health, United States of America; c Department of Surgery, Einstein Healthcare Network, United States of America

**Keywords:** Case report, Bariatric surgery, Gastric bypass, Gastric ulcers

## Abstract

**Introduction and importance:**

Roux-en-Y gastric bypass (RYGB) is one of the two most common weight loss surgeries. Surgical emergencies after gastric bypass can be complicated by devastating events that are often difficult to diagnose and manage. Perforated ulcers are a very rare complication after a RYGB.

**Case presentation:**

In this report, the diagnosis and surgical management of a 59-year-old immunosuppressed male patient who presented with late perforation of a pre-pyloric ulcer in the gastric remnant after RYGB is presented. The perforation was repaired transversely in a running horizontal mattress fashion and patched with a piece of well-vascularized omentum.

**Clinical discussion:**

This case illustrates the potential for gastric remnant ulceration, even a decade after RYGB. A high degree of suspicion for the diagnosis of perforated remnant stomach is required, especially in the absence of pneumoperitoneum and free fluid. Patient-specific factors, such as immunosuppression in this case, may blunt normal physiologic response.

**Conclusion:**

Considering the location of the ulcer in the pre-pyloric area, we caution that the typical paradigm of marginal ulceration of the gastro-jejunal anastomosis does not always apply when evaluating gastric complications after RYGB.

## Highlights

• Prepyloric Ulcer Perforation after a Roux-en-Y gastric bypass
• Immunocompromised patient may have a blunted response to a surgical complication after gastric bypass.
• Typical paradigm of marginal ulceration does not always apply when evaluating gastric complications after Roux-en-Y gastric bypass.

## 1 Introduction and importance

Roux-en-Y gastric bypass (RYGB) is one of the two most common weight loss surgeries. The goal of the procedure is to form a small gastric pouch, gastric remnant, and an anastomosis to a Roux limb of jejunum, which restricts food intake, and limits absorption. The second part of the procedure is the creation of the biliopancreatic limb which connects the rest of the stomach to the distal jejunum [1]. Complications of this procedure can either present early or late in the postoperative course. Notably, complications can be specific to the surgical approach itself, but sometimes complications can be multifactorial in nature. A recent systematic review found a total of 5 case series and 18 case reports in the literature discussing peptic ulcer disease in patients' status post RYGB [2]. We report a case of a perforated pre-pyloric ulcer in the gastric remnant over a decade after a RYGB. This case highlights a rare complication of a gastric bypass and proposes a discussion on its occurrence along with strategies proposed for early recognition and treatment. This work has been reported in line with the SCARE 2020 criteria [3].

## 2 Case presentation

**Image 1:**
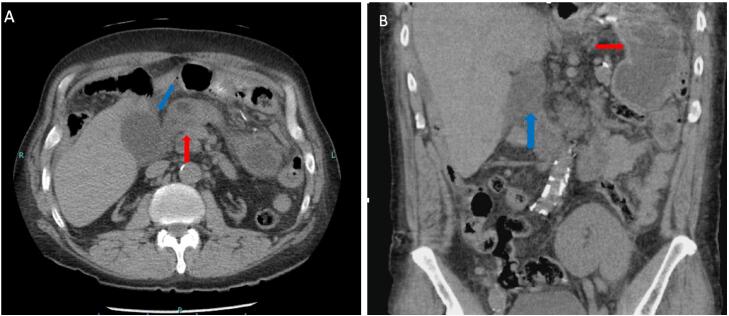
Axial (A) and Coronal (B) CT abdomen/pelvis showing distended gallbladder with wall thickening (blue arrow) and gastric remnant wall thickening with inflammatory stranding adjacent to gallbladder (red arrow). (For interpretation of the references to color in this figure legend, the reader is referred to the web version of this article.)

A 59-year-old male with a history of renal transplantation on immunosuppression (Tacrolimus) who is status post RYGB 11 years prior (prior to renal transplantation) presented to the emergency department of a tertiary teaching hospital with one week of generalized abdominal pain, nausea, vomiting, melanotic stools, and recent heavy Ibuprofen use. On examination, the patient had generalized abdominal pain, mild tachycardia but was otherwise hemodynamically stable. Blood workup was significant for leukocytosis. Computed tomography (CT) of the abdomen and pelvis depicted possible acute cholecystitis without the presence of pneumoperitoneum or free fluid. Upon further revision of the images by the senior surgery attending, gastric remnant wall thickening around the pyloric area was noticed (see Image 1).

Due to the severity of symptoms and imaging findings, the decision to perform an urgent laparoscopic exploration was made.

**Image 2:**
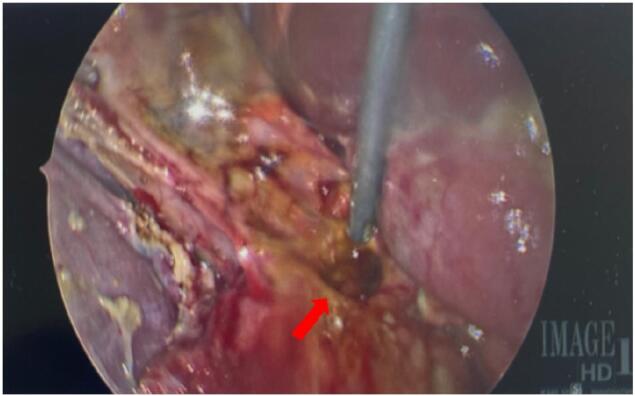
Intraoperative picture demonstrating a pre-pyloric perforation in the gastric remnant (red arrow). (For interpretation of the references to color in this figure legend, the reader is referred to the web version of this article.)

A 12-mm Hasson port was placed in the supraumbilical area and four 5-mm ports were placed in the right upper quadrant and subxiphoid area. Upon initial exploration of the right upper quadrant, bile pooling lateral to the liver was noticed. A distended, inflamed, non-perforated gallbladder was noted and due to this finding, a cholecystectomy was performed in the standard fashion. During the cholecystectomy dissection we were able to expose a well-circumscribed, 1-cm perforation of the pre-pyloric area which was draining bile (see Image 2).

The gastric pouch, Roux limb and gastrojejunal anastomosis were examined and no abnormalities were identified. The perforation was repaired transversely in a running horizontal mattress fashion with 3–0 Vicryl suture and patched with a piece of well-vascularized omentum. We elected to place a gastrostomy tube in the remnant stomach to protect from future drainage from the repaired area. Patient tolerated the procedure well.

The postoperative course was uncomplicated. The patient was discharged on postoperative day five. On two-week postoperative follow up, the patient was doing well with no evidence of on-going leakage.

## 3 Clinical discussion

Perforation of an ulcer in the gastric remnant almost a decade after a RYGB is a rare phenomenon. There have been only a few case reports and series on patients who underwent bariatric surgery with such a finding. Of those, Dai et al., described a case on a female patient with perforated ulcer with characteristic imaging findings of pneumoperitoneum on CT [4]. In these cases, Sasse et al. demonstrated that 74 % (7 cases) had a gastric perforation only [5]. However, in our case the patient did not have pneumoperitoneum on CT scan.

The etiology of gastric ulcers has been proposed in the literature. Risk factors include *H. pylori*, non-steroidal anti-inflammatory drug (NSAID), alcohol, and tobacco-based cigarettes. The influence of *H. pylori* infection in gastric ulcer disease in post RYGB patients is unclear, and international guidelines have varied regarding screening and management. Smoking has been shown to increase gastric acid secretion, hence making the mucosa prone to ulceration. NSAID use is a known risk factor for marginal ulcer formation with a reported incidence of 40 % of patients with localized marginal ulceration [3]. Proton pump inhibitors play a large role in prevention of marginal ulcers, yet the prevention of gastric remnant ulcers with proton pump inhibitor therapy have not been fully elucidated in the current literature [6,7]. In terms of perforated ulcers within the pouch, the recommended definitive management is surgical.

The pathogenesis of ulceration in patients with RYGB is not fully understood. Bjorkman et al. described one proposed mechanism involving the delay in bicarbonate secretion leading to a prolonged exposure to gastric acid [8]. Due to this prolonged exposure to gastric acid within the remnant, studies have examined the chronic histological changes within the mucosa such as atrophy and intestinal metaplasia [9]. In our opinion, the etiology in this case was multifactorial in nature.

This case also demonstrated the significance of solid organ transplantation and its corresponding complications in the gastrointestinal tract. Helderman et al., delineate the effect of certain immunosuppressive medications and their role in ulcer formation. In particular, the authors discuss how kidney transplant can introduce additional risk factors known to play a role in formation of gastric ulcers such as post dialysis gastric acid secretion and high levels of postoperative histamine and gastrin [10,11].

Notably, the classic finding of pneumoperitoneum on radiological studies is typically associated with a perforated viscus. However, reviews of the literature, particularly a review of RYGB in 2021, have suggested that pneumoperitoneum may not be apparent on diagnostic imaging [12]. As such, there should be a low threshold for further intervention in these patients. To our knowledge, this is the first described report of a RYGB patient with a perforated ulcer in the gastric remnant without imaging findings of pneumoperitoneum. Moreover, it is the first report demonstrating the formation of an ulcer in an immunocompromised patient who had undergone a RYGB over a decade ago.

Surgeons should be aware of the possibility of gastric ulcers in patients with RYGB years after the initial surgery and to consider patients who are immunocompromised at baseline may have a blunted response.

## 4 Conclusion

The presentation of a perforated prepyloric ulcer is, to a great extent, rare more than a decade after a RYGB. When evaluating complications after gastric bypasses the classic dogma may not apply in cases with immunosuppression at baseline. Finally, when imaging and clinical presentation are incongruent, it is advisable to perform a surgical exploration to assess for the possibility of complications within the RYGB structure.

## Consent

Written informed consent was obtained from the patient for publication of this case report and accompanying images. A copy of the written consent is available for review by the Editor-in-Chief of this journal on request.

## Provenance and peer review

Not commissioned, externally peer-reviewed.

## Ethical approval

This was an isolated case report done with the consent of the patient. In such case, the anonymised presentation of case report does not require a separate approvement by the ethics committee. No further research studies are being pursued.

## Funding

No external funding was available for this study.

## CRediT authorship contribution statement

Bianca Marquez, MD; conceptualization, methodology, writing original draft and final review and editing.

Emmanuel Luciano, MD; writing original draft, review and editing.

Ryan Cohen, MD; review and editing.

Christina Maser, MD; final review and editing.

Matthew Hubbard, MD; final review and editing.

## Guarantor

Bianca Marquez, MD.

## Research registration number

N/a.

## Declaration of competing interest

None declared.
